# Dry Sliding Wear Studies on Sillimanite and B_4_C Reinforced Aluminium Hybrid Composites Fabricated by Vacuum Assisted Stir Casting Process

**DOI:** 10.3390/ma16010259

**Published:** 2022-12-27

**Authors:** Manickaraj Pethuraj, Marimuthu Uthayakumar, Shanmugavel Rajesh, Mohd Shukry Abdul Majid, Sivaprakasam Rajakarunakaran, Magdalena Niemczewska-Wójcik

**Affiliations:** 1 Department of Mechanical Engineering, Saveetha School of Engineering, Saveetha Institute of Medical and Technical Sciences, Chennai 602117, Tamil Nadu, India; 2 Faculty of Mechanical Engineering, Kalasalingam Academy of Research and Education, Krishnankoil 626126, Tamil Nadu, India; 3 Faculty of Mechanical Engineering and Technology, University Malaysia Perlies, Perlies 02600, Malaysia; 4 Department of Mechanical Engineering, Ramco Institute of Technology, Rajapalayam 626117, Tamil Nadu, India; 5 Faculty of Mechanical Engineering, Cracow University of Technology, al. Jana Pawła II 37, 31-864 Cracow, Poland

**Keywords:** sillimanite, boron carbide, hybrid composites, mechanical properties, friction coefficient, wear rate, surface characteristic

## Abstract

This paper presents the results of studies to understand the influence of hybridisation on mechanical and tribological behaviour as well as dry sliding wear of aluminium metal matrix composites. Sillimanite and boron carbide (B_4_C) were used as primary and secondary reinforcements and pure aluminium was used as the matrix material. The composite was fabricated by using a vacuum assisted stir casting process. Different research instruments were used, including a scanning electron microscope with EDX spectrometer, a surface measurement device, a thermal image analyser, as well as a tribotester. The results show that tensile, impact strength and hardness of the hybridised composites are superior (a step ahead) than unreinforced and primary composites. The wear behaviour of the fabricated specimens was tested for the dry sliding wear behaviour under the load range of 10–50 N with the steps of 20 N for the sliding velocities 0.75, 1.5 and 2.25 m/s over a distance of 1000 m. The wear rate increased with load and decreased as the wt.% of reinforcement increased. The wear rate of the composite with 10 wt.% Al_2_SiO_5_ was approximately 44% lower than that of the composite with 5 wt.% Al_2_SiO_5_. The same dependence was noted for hybrid composite (5 wt.% Al_2_SiO_5_ + 5 wt.% B_4_C)—the wear rate was approximately 50.8% lower than that of the composite with 5 wt.% Al_2_SiO_5_ under the same test condition. The friction coefficient decreased as the weight percentage of the reinforcement (Al_2_SiO_5_ and B_4_C) increased due to the uniform distribution of the reinforcement on the surface of the composites. The main wear mechanism of the studied materials was abrasion wear. The wear mechanism of the composite had tribochemical type. It involved the oxidation and transfer of the material, which formed protective tribolayers ensuring an additional sliding process. The mechanism that played the main role in the wear process of the composites was a combination of abrasive, adhesive and oxidative wear.

## 1. Introduction

The major global industrial sectors, such as automobile, aerospace, military and nuclear, are experiencing the advantage of using particulate reinforced Aluminium Metal Matrix Composite (AMMC) because of its exceptional mechanical and tribological property [1]. Particulate reinforced composites are effectively produced by a variety of methods, such as powder metallurgy, plasma spraying, spray atomisation and co-deposition, stir casting and squeeze casting. Among the aforementioned, an efficient method for fabricating the composite is the stir casting process. It is one of the inexpensive methods that provide options to adjust the process conditions and remains one of the most widely used methods in industry [2]. The material science community is looking for freedom in designing new kinds of materials [3], and this freedom can be fulfilled by hybridisation techniques. This hybridisation of materials provides high strength, stiffness and with low density. The hybrid materials are also called second generation composites materials, since they act as a substitute for the single reinforced composites [4]. The conventional materials and single reinforced composite materials have insufficient mechanical properties therefore their use is limited. Technical parts made of the second-generation composites manufactured by the hybridisation techniques cater to the needs of different industries [5].

The comfort distribution of primary and secondary reinforcement enables the fabrication of hybrid composite materials. In general, metallic materials are reinforced with ceramic materials because they possess superior strength to any of the other reinforcements [6,7]. Aluminium is one such metallic material and it possesses good mechanical properties, but affects with low elastic modulus and wear resistance properties. As a consequence, aluminium and its alloys experience deflection during the application of load and are subject to heavy wear in the case of tribological application. One strategy is stiffening the materials with the addition of ceramic materials, which provides better elastic modulus and wear resistance to aluminium and its alloys [8,9].

The most commonly used ceramic reinforcements are silicon carbide (SiC) and alumina oxide (Al_2_O_3_). The density of these ceramic reinforcements is 3.18 g/cm^3^ and 3.9 g/cm^3^, respectively, whereas aluminium is 2.7 g/cm^3^. The addition of these reinforcements over the matrix materials increases the stiffness, strength, hardness and density of the composites [10,11]. The changes in mechanical property helps the composites to exhibit superior wear resistance to the monolithic aluminium alloy. Another reason for increasing wear resistance is the shifting of the wear mechanism. Adhesion is the major wear mechanism when there is metal to conduct, whereas in the case of metallic materials reinforced with ceramic materials, the wear phenomenon is changed to oxidative—adhesive wear mechanism [12]. The type of oxidative—adhesive wear mechanism may be varied with the kind of matrix materials, type, size and shape of the primary and secondary reinforcement, including the fabrication methods. Compared to cast iron, aluminium MMC has a stronger resistance to wear. By using MMC, an engine block’s overall weight is lowered by 20%. Additionally, because aluminium MMC has better thermal conductivity, it can operate at lower temperatures for longer periods of time. Because the cylinder barrel is thinner than the one made of cast iron, the precise engine’s operating volume may be increased without having to modify it [13].

Ceramic materials with different forms are reinforced with metallic phase materials on which particulate reinforced composites are finding application in automotive industries for making brake rotor systems, piston cylinder heads, piston rings, liners and pump bodies [14]. An attempt has been made by various researchers to fabricate aluminium-based composites using different ceramic materials. In addition to SiC and Al_2_O_3_, the most widely used other types of reinforcement are boron carbide (B_4_C) and graphite. It is understood from the literature that the cost of the B_4_C is comparatively more expensive than the other reinforcements, such as SiC and Al_2_O_3_. Because of the higher cost and limited import facility of the B_4_C reinforcement, developing countries find it difficult to develop particulate reinforced materials. If these reinforcements are used as secondary reinforcement material in aluminium, it may reduce the cost of the composites without losing the required properties. The addition of secondary reinforcement in the matrix materials will reduce the density of the composites if the density of the secondary reinforcement is low and with a minimum percentage of reinforcement content. It is also possible to obtain better ductility and toughness property of the composite if a suitable primary/secondary reinforcement will be added for making the hybrid composite. In addition to the above, the cost of the composites becomes low once the primary reinforcements are available at low cost [15].

Recently, an attempt has been made by researchers to make use of agricultural and industrial remainder materials, such as red mud, fly ash, and rice husk as secondary reinforcements to fabricate hybrid composites to reduce the cost [16,17]. However, the properties of the composites depend upon the processing route, nature of the matrix, and primary and secondary reinforcement materials. Mostly, the performance of the hybrid composites is decided by the reinforcement shape, size, modulus of elasticity, hardness, binding of the reinforcement over the matrix, followed by the processing route and its parameters. Most studies are carried out by reinforcing with SiC and Al_2_O_3_ (hybridisation), and it is found that fabricated composites are good in strength and perceived stiffness, but that this occurs at the expense of ductility and fracture toughness. These two important properties prevent the failure from the in-service stress and impact load. This could be another reason for synthesising the composites with secondary reinforcements.

The most commonly used secondary reinforcements are fly ash, graphite and B_4_C, along with SiC and Al_2_O_3_. The addition of B_4_C theoretically provides better elastic modulus to the composites even at a lower percentage of reinforcement (≤10%) [7]. The application of B_4_C reinforced composites is unlimited in nuclear reactors—as neuron absorbers, armour plates, and computer hard disk substrates [18]. The other reinforcements are sillimanite [19,20], rutile [21], zircon [22] and garnet, which are abundantly available in the coastal part of India. To reduce the cost of the composites, the aforementioned materials are used by various researchers and, in addition, they have made an attempt to understand the wear behaviour of these composites. The density and bulk modulus of the sillimanite is 3.240 g/cm^3^ and 171 GPa. Although the density of the reinforcement is high when compared to aluminium and B_4_C, it possesses excellent wettability with aluminium matrix, high hardness, low coefficient of thermal expansion, excellent high thermal stability and non-reactive nature [23].

Singh et al. [24] attempted to use the natural reinforcement for making the sillimanite-based reinforced aluminium composites through solidification techniques. The fabricated composite was superior in mechanical and tribological properties to aluminium alloy. The hardness of the sillimanite reinforced composites was 85 HV, whereas the base aluminium alloy was 57 HV. In a continuation of the previous studies, the experimental results of Singh et al. [25] of the two-body wear behaviour, sillimanite reinforced aluminium composite revealed better wear resistance with the addition of 25 µm size particulates than for the matrix alloy. With the addition of 200 µm size particulates, the results were worse than for the matrix alloy. Comparatively, a better result was obtained when the size of the particulates was approximately 100 µm.

The effect of particulate size on the wear resistance property of sillimanite reinforced composite fabricated by stir casting process was studied by Sharma et al. [23]. Three different particulate sizes were used to fabricate and conduct the wear test. A small sized particulate in the range of 1–20 µm provided better wear resistance, which was 55% higher when compared to the monolithic aluminium alloy. The other particulates sizes were 32–50 and 75–106 µm that did not have that much influence on the wear rate. Lee et al. fabricated the Al6061/B_4_C composite by varying the wt.% from 5 to 20%, using the stir casting process accompanied by hot rolling. The wear studies were conducted by varying the load at 5N and 20N. It was reported that the friction coefficient decreased with the addition of the B_4_C particulates till 10 wt.% and it started to increase compared to the Al6061. It was also reported that the hardness increased with the addition of B_4_C particulates. The main wear mechanism reported was delamination [26].

Hardness and impact strength of the B_4_C reinforced AMMC was produced by powder metallurgy method, which was studied by Topcu et al. [27]. The addition of B_4_C decreased the impact strength and increased the hardness of the composite. Thus, the fabricated composite had a hardness value raised from 25.8 to 82.1 HV for 20 wt.% in the reinforced composites. To achieve this hardness, the sintering temperature was maintained to 650 °C. The impact energy of the composite was reduced from 42 kJ/m^2^ to 9 kJ/m^2^ for the same fabrication condition and weight percentage. The effect of B_4_C on tribological behaviour of aluminium reinforced composites was studied to understand the reason for less wear in the composites when compared to the base matrix alloy. Baradeswaran and Elaya Perumal [8] observed that the insulation layer between the metal-to-metal contact, oxygen, and iron between the metal surface could be the reasons for forming a mechanically mixed layer (MML).

Singh and Goyal [28] studied the wear resistance properties of Al-SiC-B_4_C reinforced composites. The hardness of the composites was raised up to (7.5 wt.% SiC and 7.5 wt.% B_4_C) 15 wt.% of reinforcement and tended to decline. The reason for the decrease in the hardness value was due to the cluster formation of the reinforcement particulates in the matrix, which in turn lowered the densities. It was also understood that sliding distance was one of the important factors affecting the wear resistance property, followed by sliding speed and load.

Soft reinforcements are also used as secondary reinforcements for providing solid lubrication property to the composites, which in turn gives better tribological properties to the hybrid composites. Monikondan et al. [29] fabricated Al-B_4_C-MoS_2_ hybrid composites via the stir casting route. The addition of MoS_2_ on Al-B_4_C reduced the fracture toughness and hardness of the composites, but it provided better wear resistance than the Al-B_4_C composites. Baradeswaran et al. [30] used graphite as a secondary reinforcement to fabricate Al-B_4_C composites through the stir casting route. Two different series aluminium alloy, namely AA6061 and AA7075, were used as the matrix material, B_4_C and graphite were used as the primary and secondary reinforcement, and these were subsequently tested. It was noted that the tensile strength and hardness of both composites increased. Ramnath et al. [31] fabricated aluminium hybrid composites using B_4_C and Al_2_O_3_ as reinforcements using the stir casting process. Pranavi et al. [32] fabricated an aluminium hybrid composite by varying the weight percentage of B_4_C and Al_2_O_3_ in equal proportions with Al 5059. The results of the studies reported that the mechanical properties and surface properties improved with the increase in wt.% of the ceramic reinforcement. Also, the specific wear rate decreased with the increase in load of 30 N, and the friction coefficient decreased with the increase in the wt.% of reinforcement for the same load. Subramanian et al. [33] fabricated Al7068/Si_3_N_4_/BN composite using the stir casting method. The wear studies were planned by the Taguchi method using applied load, sliding velocity and sliding distance as the process parameters. It was noted that the composite material reinforced with 10 wt.% Si_3_N_4_ and 5 wt.% BN was determined to have the lowest wear rate. To examine the impact of the process factors on wear rate and friction coefficient, analysis of variance (ANOVA) was used. According to the results of the ANOVA test, load had a greater percentage contribution (29.73%) to friction coefficient than Si_3_N_4_ weight fraction (36.60%) did to the wear rate [33].

It is understood from the literature that there are reports on hybrid composites and their tribological properties, but there are no systematic studies on sillimanite—B_4_C reinforced AMMC. Therefore, the paper aims to present the fabricated hybrid composite using a vacuum assisted stir casting setup with the results of the studies and discussion.

## 2. Materials and Methods

### 2.1. Materials

**Figure 1:**
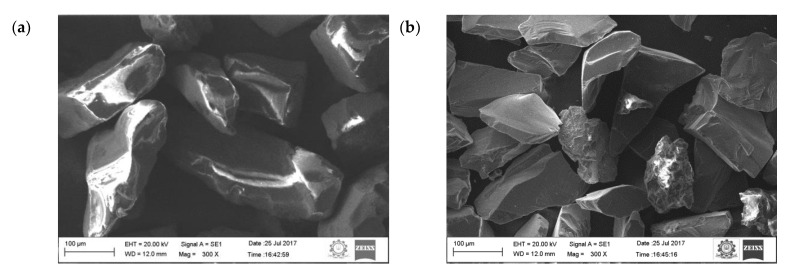
SEM image of particles: (**a**) sillimanite, (**b**) B_4_C.

In this work, 99.5% pure aluminium in the form of an ingot was used as the matrix material. The required quantity of the pure aluminium was bought from Coimbatore Metal Mart (Coimbatore, India). Sillimanite and boron carbide (B_4_C) were used as primary and secondary reinforcements in the form of particulates. The particulates of the sillimanite (as received) and boron carbide are shown in Figure 1. Average particulate size of both primary and secondary reinforcement is in the range of 55 to 80 µm.

Sillimanite is the compound of aluminium, silicon and oxygen having the chemical formula of Al_2_SiO_5_. Table 1 shows the details of the chemical composition of the primary reinforcement. Sillimanite is available in different colours, in the southern part of India mostly in a brown colour. Sillimanite is chemically inert and stable, therefore it is used as a primary reinforcement material (purchased from VV Minerals, Tirunelveli, India).

Boron carbide (B_4_C) is one of the promising reinforcement materials because of its excellent properties, which include high strength and low density. Moreover, it is the third hardest material existing on the planet after diamond and cubic boron nitride (cBN). The toughness of the B_4_C is very close to diamond and chemically stable even at an elevated temperature. In addition, it possesses excellent neutron absorption capability. The properties of aluminium, primary and secondary reinforcement are listed in Table 2.

### 2.2. Fabrication of the Composites

**Figure 2:**
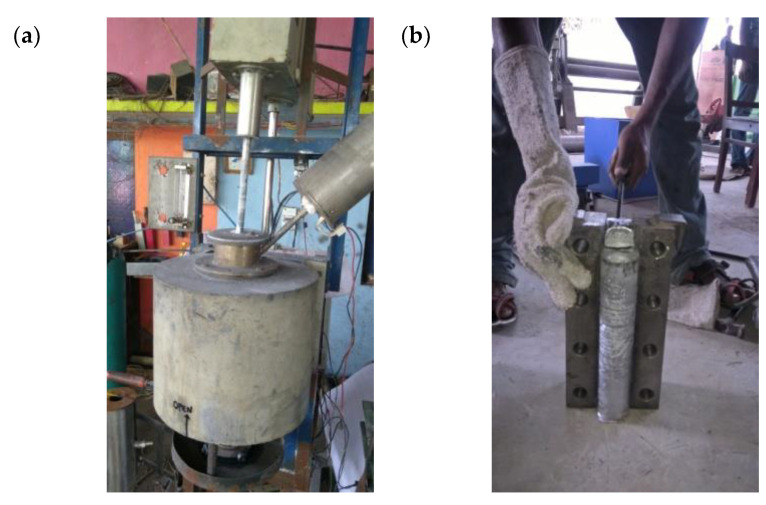
Equipment setup: (**a**) vacuum casting setup (**b**) fabricated composites.

The aluminium material was sized to the required dimension and placed in a graphite crucible. The required quantities of aluminium were melted in the electrical furnace and heated up to 750 °C and the same temperature was maintained up to 60 min. Similarly, the required quantities of sillimanite particulates were taken in the graphite crucible and heated with electrical induction furnace up to 1000 °C. Preheating of the reinforcements provided better thermal equilibrium condition during the mixing of reinforcement with the matrix material. The preheated reinforcement was slowly added with the matrix materials, and stirring speed was maintained between 500–550 rpm. To increase the wettability, 2 wt.% of magnesium was also added to the molten metal. The same procedure was adopted to make pure aluminium, 5 and 10 wt.% of sillimanite, as well as hybrid composites (5 wt.% of sillimanite + 5 wt.% of B_4_C). In the case of the hybrid composites, to avoid agglomeration in one reinforcement in one part of the matrix, the primary and secondary reinforcement materials were mixed uniformly before pouring into the molten metal. The addition of two reinforcements led to an increase in porosity; to minimise the porosity, a special arrangement was made to remove the unwanted gases formed during the solidification process. The special arrangement consisted of a metal die attached with a vacuum pump; while pouring the molten metal, it evacuated the unwanted gases and helped the fabrication of the composites become flawless. Figure 2 shows the equipment setup used for the fabrication of the composites. The required number of samples was prepared in the form of a round rod and plate; the size of the round rod was ø 150 × 300 mm.

The following characterisation and testing was conducted to study the properties of the fabricated composite. The fabricated specimen was cut into a longitudinal cross-section to understand the distribution of sillimanite and B_4_C particulates over the aluminium material. The cut section of the composite was gently ground using different SiC grid paper followed by diamond polishing. The polished specimen was further metallographically polished using Keller reagent; the reagent was prepared as per the standards described in [30]. Surface morphology (microstructure) of the composites and its hybrids was investigated by a Scanning Electron Microscope JEOL JSM–6480 with LV type filed emission using appropriate accelerating voltage.

SEM–EDX was used to identify the existence of the primary and secondary reinforcements. The density of the fabricated composites was measured by the Archimedes principle according to the standard ASTM E9-89a. The ratio of length to diameter of the cylindrical specimen was maintained as 2:1. The theoretical density ($\rho_{c_{t}}$) of the specimen was estimated by the expression given by Agarwal and Broutman [34]. Equations (1) and (2) were employed to determine the density of specimens.

(1) $$\rho_{c_{a}}=\frac{1}{\left(\frac{w_{m}}{\rho_{m}}\right)+\left(\frac{w_{r}}{\rho_{r}}\right)}$$

(2) $$\rho_{c_{a}}=\frac{1}{\left(\frac{w_{m}}{\rho_{m}}\right)+\left(\frac{w_{r_{1}}}{\rho_{r_{1}}}\right)+\left(\frac{w_{r_{2}}}{\rho_{r_{2}}}\right)}$$

where $\rho_{m}$ and $w_{m}$ are density and weight of the aluminium materials; $\;\rho_{r_{1}},\;\rho_{r_{2}}$ and $w_{r_{1}},$ $w_{r_{2}}$ density and weight of the primary and secondary reinforcement materials. $\rho_{c_{t}},\;\rho_{c_{a}}$ was the theoretical and actual density of the specimen. Actual density of the specimen was measured using the Archimedes principle. Void fraction of the composites was measured by the following expression given in Equation (3):

(3) $$V_{f}=\frac{\rho_{c_{a}-}\rho_{c_{t}}}{\rho_{c_{a}}}\times 100$$

where $V_{f}\;$ was the void fraction of the specimen stated as a percentage. The hardness of the composite was measured using a Vickers Micro Hardness testing machine (according to the standard ASTM E92) and diamond intender. The hardness value of the composites and hybrid composites was reported by taking the average value of five tests.

The following expression given by Equation (4) was used to compute the microhardness of the composites and its hybrids. The 4.903 N load was applied on the specimen for 10 s:

(4) $$H_{V}=0.1889\;\frac{F}{L^{2}}\;and\;L=\frac{X+Y}{2}$$

where *X*, *Y*, and *L* were the horizontal length, vertical length and length of the diagonal. Load *F* was applied in N. ASTM standard B577M was used to compute the tensile strength of the primary and secondary reinforced composite specimen (Tensile Testing Machine). ASTM standard A370 was used to measure the impact strength of the specimen (Impact Strength Machine). The testing was repeated—the average value was taken for analysis.

### 2.3. Characterisation of Dry Sliding Wear

Tribotester with friction pair type ‘pin-on-disc’ (Ducom, model TR-20) was used to conduct the dry sliding wear test on the composite specimen, for varying load (10 to 50 N). The sliding velocity of the test was also varied between 0.75 m/s, 1.5 m/s and 2.25 m/s. The sliding distance for the entire test was fixed as constant (1000 m). The surface topography was measured with the use of the surface measurement device (Gwyddion). ASTM G99 standard was followed to perform the wear studies, the diameter and height of pin was maintained as 10 × 30 mm.

Wear tests of the composite and its hybrids were conducted under dry sliding condition against the EN32 steel disc having the hardness of 64 HRC at ambient environmental condition. The initial and final weight of the composites and its hybrids were measured using an electronic weigh balance with an accuracy of 0.0001 mg. The mass loss of the specimen was used to compute its specific wear rate for varying load and sliding condition. The friction coefficient *μ* was calculated based on the normal and tangential load.

After the wear test, each worn surface of the composite was studied using both surface measurement device and SEM. The thermal image analyser was exactly perpendicular to the contact surface of the pin and disc for taking the thermal images. The captured image was used to identify the hot spot of the pin and disc and to correlate with the vibration signal.

## 3. Results and Discussion

### 3.1. Surface Morphology

**Figure 3:**
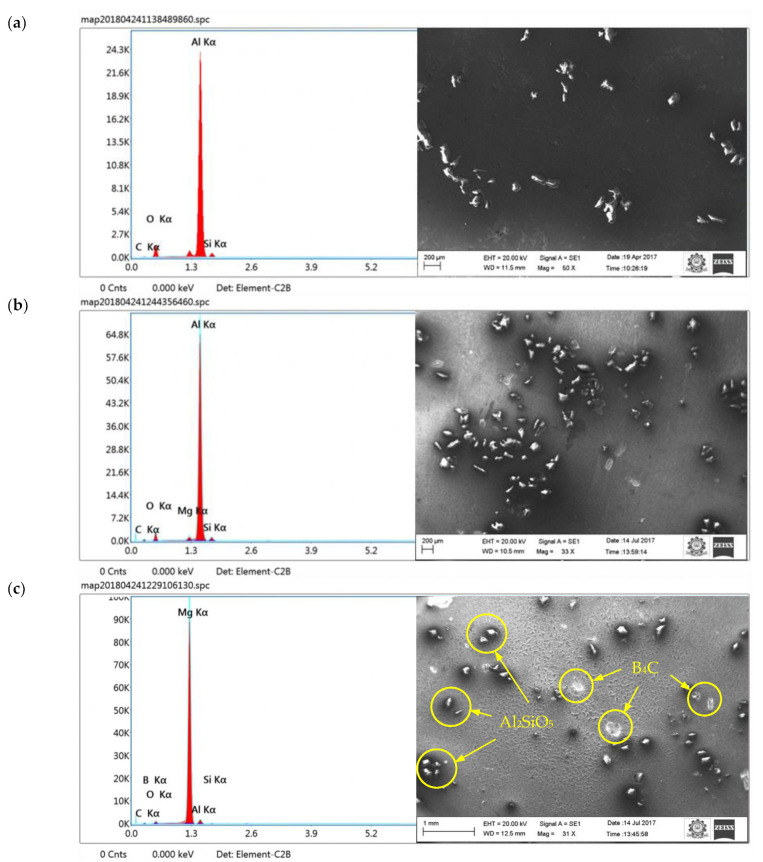
Surface morphology (SEM images) and EDX image: (**a**) 5 wt.% (**b**) 10 wt.% [35] and (**c**) hybrid composites.

Figure 3a,b shows the surface morphology (microstructure)—SEM images and the EDX images of the primary reinforced composites, reinforced with 5 and 10 wt.%. Figure 3c shows the existence of primary and secondary reinforcement particulates in the specimen (5% of sillimanite and B_4_C). It was understood that the primary and secondary reinforcements were uniformly distributed throughout the aluminium material. The larger magnification of hybrid composites on the SEM image show better interfacing between the matrix and reinforcement, either without or reduced porosity. In the case of hybrid composites, the primary and secondary reinforcements were mixed thoroughly, and the secondary reinforcement was well placed between the primary reinforcements. Correct stirring speed selection and uniform mixing of the reinforcement was monitored to ensure proper mixing of the primary and secondary reinforcements in the matrix materials, otherwise, due to the densities’ difference between the reinforcements, there was a possibility of lighter reinforcements settling at the top of the molten metal, even though the viscosity of the molten condition was closer to 1 kg/m^3^. Thus, it was mandatory to have stirring speed and time and to allow the molten metal to enter into the die.

The reinforcement size is one of the influencing factors in the distribution of the reinforcement over the aluminium material [36]. In this case, the size and shape of the primary and secondary reinforcement was also closer and similar. The thermal and heat conductivity of both reinforcements were similar, therefore both could receive more or less the same temperature, which in turn avoided clustering of the reinforcements in one place. In addition, the proper fluidity condition and sufficient solidification rate were maintained to achieve correct distribution of the reinforcement particulate.

In Figure 3a,b, the EDX images show the existence of aluminium, alumina, silicon, sillimanite and aluminium silicate in the primary reinforced composites. Figure 3c shows the EDX images for the hybrid composites, the existence of B_4_C, along with primary reinforcement chemicals. The properties of the composite specimen were altered by the interfacial reaction and secondary phase formation. In the case of B_4_C, it did not undergo reaction, thus the strength and stiffness of the composites were improved.

### 3.2. Density, Hardness, Tensile Strength and Impact Strength

**Figure 4:**
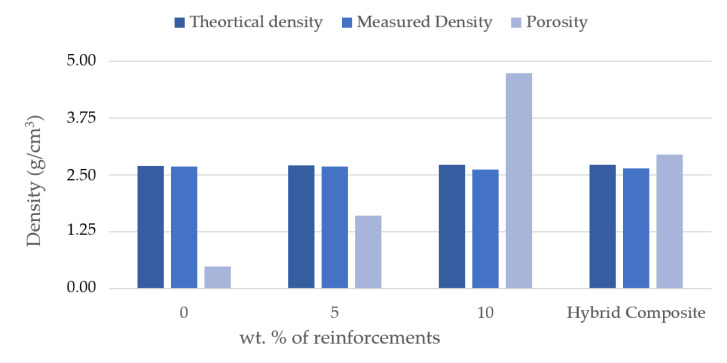
Density (theoretical and measured) and porosity of the specimen.

Figure 4 shows the difference in densities of the composites and their hybrids. In general, the addition of ceramic material over the aluminium matrix material increases the density, unless the ceramic reinforcements density is less than the aluminium and its alloy. In hybrid composites, the density will be lower if the density of the secondary reinforcement is lower [37]. In this case, the secondary reinforcement was lower than the matrix and primary reinforcement materials; it was obvious that the density of the hybrid composites was less. Another reason could be a perfect interfacing between the reinforcement and matrix materials. It was also observed that the difference in density among the composites and its hybrids was less than 1% only. It was reported in the literature that augment in wt.% of reinforcement steer increases the porosity, unless the process parameters remain under control [38]. Generally, the porosity of the composites increased as the reinforcement content increased; in 5 and 10 wt.% composite it remained less than 5%, and in the case of hybrid composites was even lower than 3%, which was achieved by stir casting followed by a vacuum casting process.

**Figure 5:**
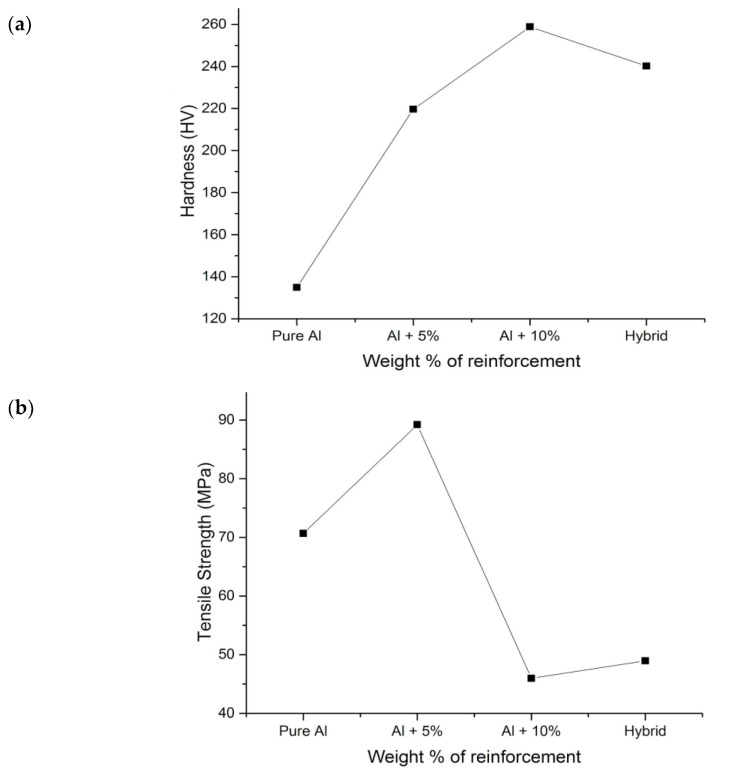

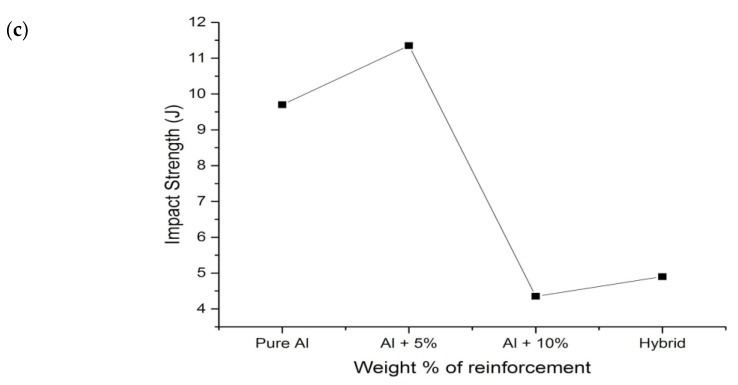
Mechanical properties of the composites and its hybrids: (**a**) hardness, (**b**) tensile strength, (**c**) impact strength.

Figure 5a shows the discrepancy of hardness based on the wt.% of the primary reinforcements and secondary reinforcements. It was noted that the hardness of the specimen increased when compared to the matrix materials. The reason for the increase in hardness was owing to the existence of comparatively hard reinforcement particulates in the composites. Sillimanite and B_4_C were relatively harder elements than the matrix materials. The material structure (hard particulates) acted as barrier to the motion along the slip plane, owing to which the composite was not easily deformed by the influence of the indenter on the material [30]. The local plastic deformation of the aluminium material was constrained due to the thermal stress presence of sillimanite and B_4_C. In the case of hybrid composites, they were slightly lesser than 10 wt.% reinforced composites, and the interaction between the primary and secondary reinforcements could be the reason for the clustering of sillimanite and B_4_C.

Figure 5b shows the effect of primary and secondary reinforcement on tensile strength of the specimen with varying wt.%. It was understood from the graphical representation that incorporation of particulates increases the tensile strength of the specimen to a certain extent. From the microstructural observation, it was evident that the reinforcements were uniformly scattered over the aluminium; it was achieved by properly preheating and stirring the molten metal. The strong interface between the Al and reinforcement could be one of the reasons for the increase in tensile strength. That interface acted as barrier to the plastic formation and resistance to the load. A similar result was also noted by Milan and Bowen [39]. The increase in tensile strength was also attributed to the thermal stress generated because of the thermal mismatch between the reinforcements and the aluminium materials. Aluminium has a higher thermal coefficient, whereas the reinforcements are less than that of aluminium, due to which thermal stress is established during the solidification and cooling process. Thus, the generated thermal stress during the fabrication of composites acts as barrier to the applied load and increases the density of the dislocation, which in turn increases the strength of the composites [40].

Figure 5c presents the variation in the impact strength of the specimen for varying wt.% of the reinforcements. Energy observed by the 5 wt.% primary reinforced composite was superior to the other composites and its hybrids. The increase in impact strength was due to the reduction in the porosity [25]. Moreover, it was observed that the porosity of the monolithic aluminium and 5 wt.% is less than the 10 wt.% reinforced composites.

### 3.3. Wear Behaviour and Worn Surface of the Composites and Their Hybrids

**Figure 6:**
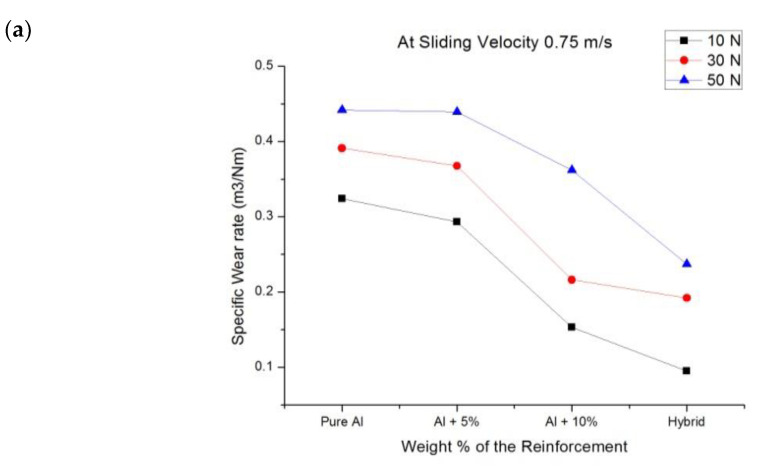

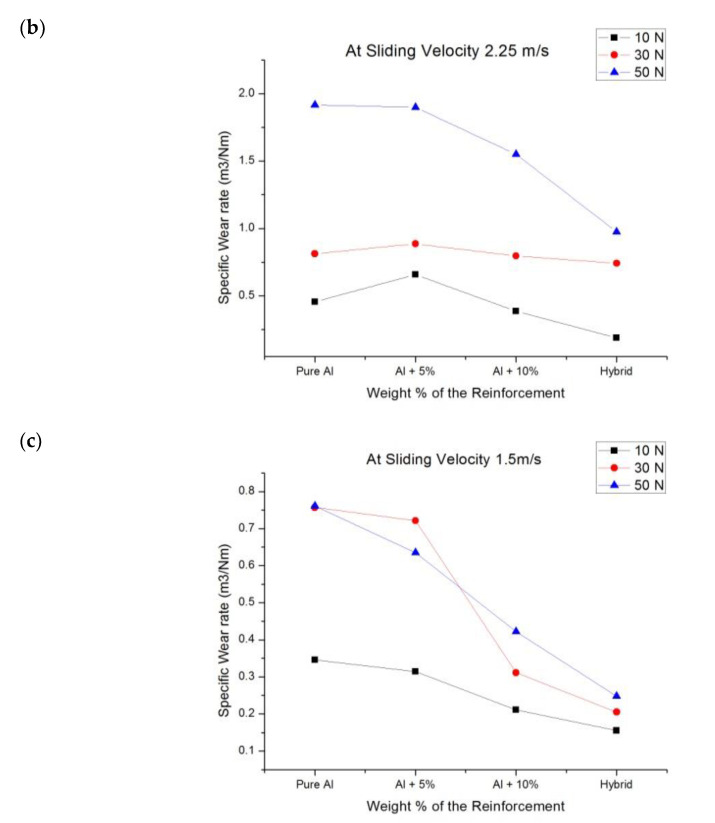
Wear rate of the composites at sliding velocity: (**a**) 0.75 m/s, (**b**) 1.5 m/s (**c**) 2.25 m/s.

Figure 6 shows the influence of normal load on specific wear rate of the different specimens at different sliding velocities. It was noted that the addition of particulates in the aluminium matrix reduces the specific wear rate of the composites. At 0.75 m/s sliding velocity, the hybrid composites experienced minimum specific wear rate. The increase in the applied load significantly increases the specific wear rate; a similar kind of trend was also observed in other sliding velocities.

The specific wear rate and friction coefficient increased with the rise in sliding velocity. The surface topography (surface roughness) of the composites was also measured after each experiment. It was noted that the increase in sliding velocity and applied load adversely influenced the surface roughness of the composites.

The reason for the higher specific rate of 5 wt.% reinforced composite at 2.25 mm/s sliding velocity (Figure 6b) is that as the temperature increased, the stable oxide layer between the matrix and reinforcement was broken. The overlapping was due to the increase in specific wear rate of Al +5 wt.% of composite at 30 N load compared to that of 50 N load (Figure 6c), as the interface between the matrix and reinforcement particulates was strong and the oxide layer was stable enough to withstand the load.

**Figure 7:**
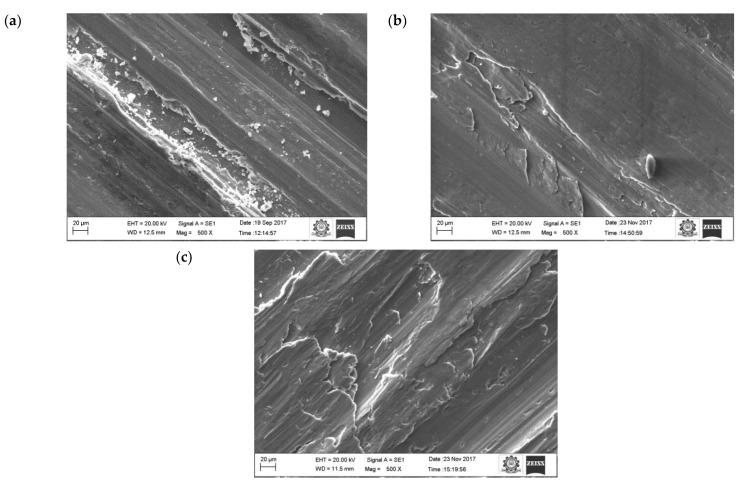
Worn surface morphology (SEM images) of the pure aluminum: (**a**) 0.75 m/s, (**b**) 1.5 m/s (**c**) 2.25 m/s.

Figure 7 shows the morphology of the worn surface of the aluminium material at 10 N load for different sliding velocities (0.75. 1.5 and 2.25 m/s). The wear mechanism of pure aluminium at sliding velocity (0.75, 1.5 and 2.25 m/s) and applied load (10, 30, 50 N) were more or less constant. This was attributed to the formation of a stable oxide layer between the counter surface and pin material. The existence of oxides content was seen on the EDX image.

The oxide layers were strong and stable, whilst at the same time unstable at higher loads. At higher loads, the increase in temperature between the counter surface and pin material broke some of the oxide film layer, which resulted in increased specific wear rate. A similar result was also observed by Colas et al. [41]. In their case, it was also observed that at maximum load the specific wear rate of the composites was higher. At the beginning, the oxide layer was very thin and propagated as a strong layer while sliding distance was increased gradually. The oxide layer was harder than the base surface and pin material due to the strain hardening effect, therefore it started to cut the surface of the pin. Due to that effect, the worn surface consisted of numerous ridges and long grooves running along the direction of the slides. It was noted that this was because of the abrasion wear mechanism. It was also visible on the worn surface of the aluminium materials (Figure 7a).

**Figure 8:**
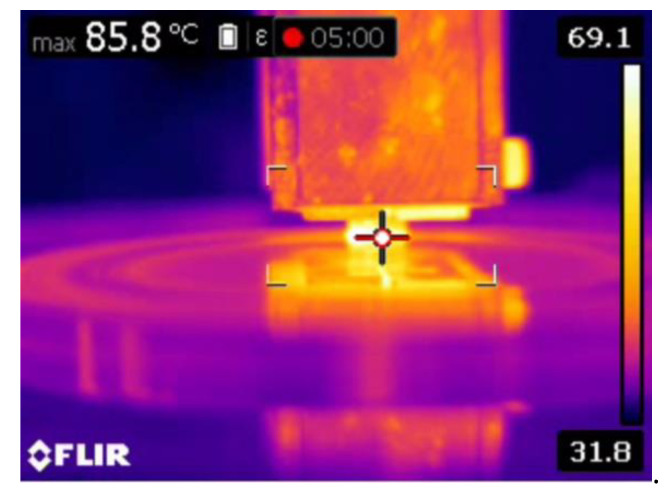
Temperature of the pure aluminium pin at 50 N loads at 5 min.

It is also evident that the thermal image (Figure 8) was taken at a higher sliding speed and load. At a higher sliding velocity and load, the temperature between the counter surface and pin material was recorded as maximum, which could be the reason for the formation of the increased number of grooves at a higher sliding velocity and load. The formation of the grooves was due to the emergence of small craters on the surface.

**Figure 9:**
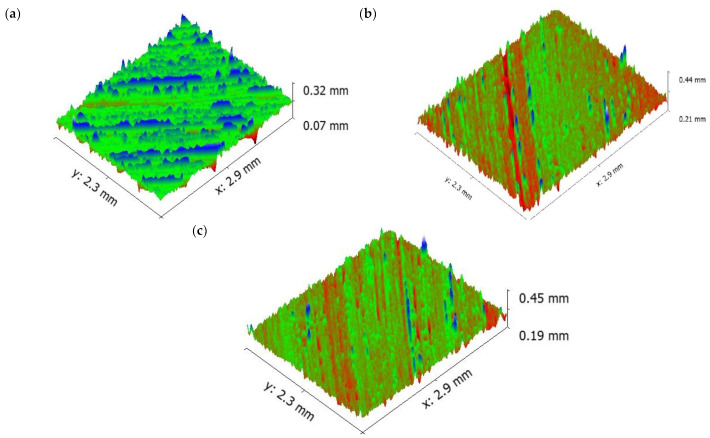
Worn surface topography of the pure aluminium: (**a**) 0.75 m/s (**b**) 1.5 m/s (**c**) 2.25 m/s.

Increasing the sliding speed increased the number of ridges and grooves on surface of the aluminium (Figure 9). It is evident that from the increase in applied load, there was an increase in the wear rate of the specimen because of the abrasion wear as well as the formation of small craters on the surface. SEM images of the pure aluminium (Figure 7) reveal that the depth and width of the groove is deep at higher load and sliding velocity conditions (50 N load and sliding velocity 2.25 m/s). The reason for the deep groove could be because of the hard-protruding point of the counter surface pressed into the pin material (matrix material) due to which it restricted the movement of the pin over the disc. It caused the extreme shearing strain over the matrix material, which led to the formation of wide, deep grooves and sometimes severe plastic deformation. The surface topography of the pure aluminium shows that the surface roughness of the specimen (values visible on the vertical axis) increased whilst sliding velocity increased (Figure 9).

**Figure 10:**
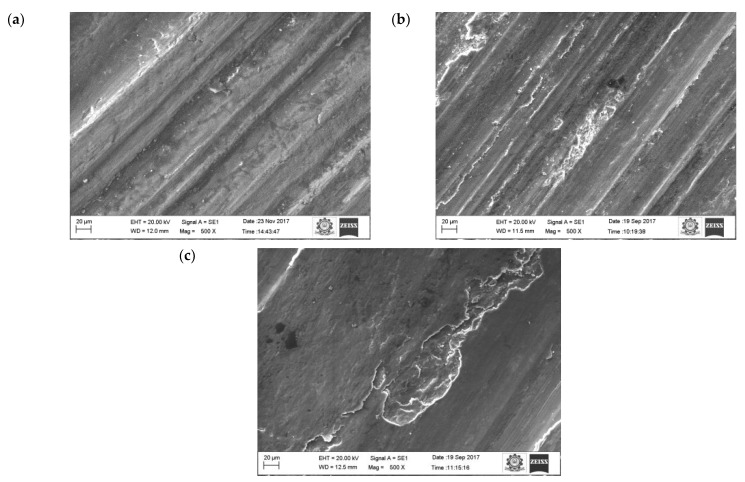
Worn surface morphology (SEM images) of the 5 wt.% composite: (**a**) 0.75 m/s (**b**) 1.5 m/s (**c**) 2.25 m/s.

It is understood from Figure 6 that the addition of 5 wt.% reinforcement reduced the specific wear rate of the specimens. The reduction in specific wear rate was attributed to the reduction in contact area between the counter surface and pin material. The reason for the reduction in contact area was due to the contact of the protruding primary reinforcement over the material of the counter surface. The protruding particulates over the pin were acting as load bearing members, thereby reducing the contact with the matrix materials; it is the formation of an oxide layer between any kinds of metal. This phenomenon led to the formation of an oxide layer between the counter surface and pin material. In such a case, the formation of oxides was not only from the pin material but also from the material of the counter surface. The material of the counter surface contained ferric content and reacted with the environment, forming ferric oxide, which served along with alumina oxide to form a strong mechanical mixed layer. The mechanically mixed layer was stronger than the pure aluminium oxide layer. This was confirmed by measuring the microhardness of the worn surface composites and aluminium matrix materials. Such behaviour was in good agreement with the work of Mathalai Sundaram et al. [42]. The SEM image of the 5 wt.% reinforced composite revealed that even at higher loads, the failure due to plastic deformation was less. In addition, the temperature between the counter surface and pin material was slightly lower than the pure aluminium; it showed the existence of a strong oxide layer between the matrix and pin materials. The strong oxide layer was able to withstand the applied load for a longer time than the pure aluminium. It could be one of the reasons for a reduction in the specific wear rate. Another reason could be that most of the shearing strain was observed by the projected primary reinforcement particulates. At a higher load—similar to pure aluminium—the formation of a deep groove was shown (Figure 10) in the 5 wt.% reinforced composite, but the depth of the groove was comparatively less than the pure aluminium [43].

**Figure 11:**
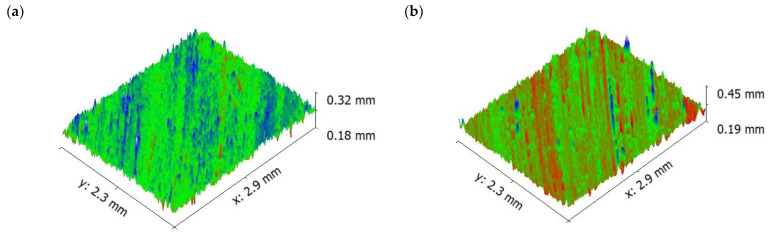

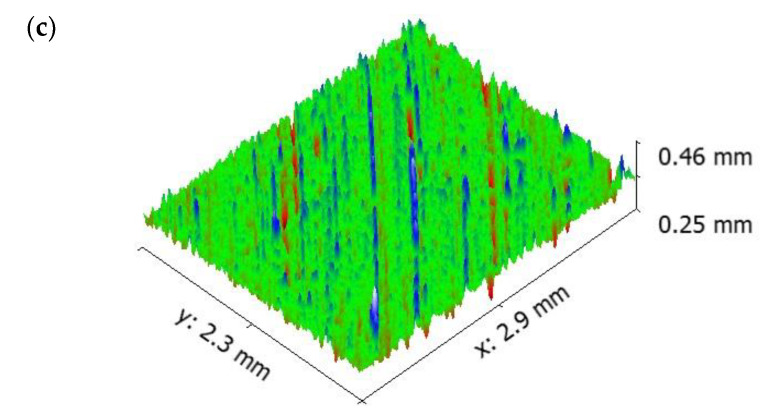
Worn surface topography of the 5 wt.% composite: (**a**) 0.75 m/s (**b**) 1.5 m/s (**c**) 2.25 m/s.

It is evident from Figure 11 that the depth of groove is low even at higher sliding velocity. The surface topography of the 5 wt.% composite shows that the surface roughness of the specimen (values visible on the vertical axis) increased as sliding velocity increased, similar as for pure aluminium (Figure 9).

**Figure 12:**
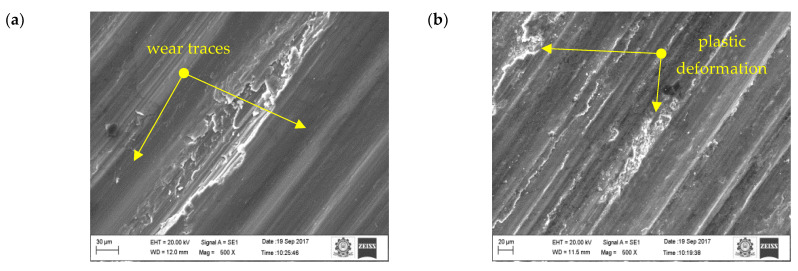

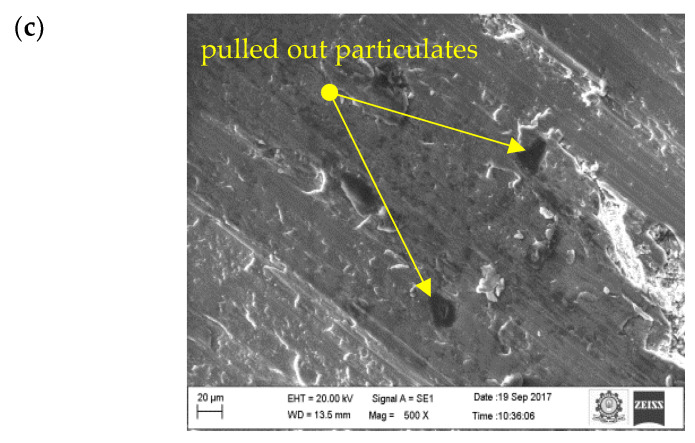
Worn surface morphology (SEM images) of the 10 wt.% composite: (**a**) 0.75 m/s, (**b**) 1.5 m/s, (**c**) 2.25 m/s.

**Figure 13:**
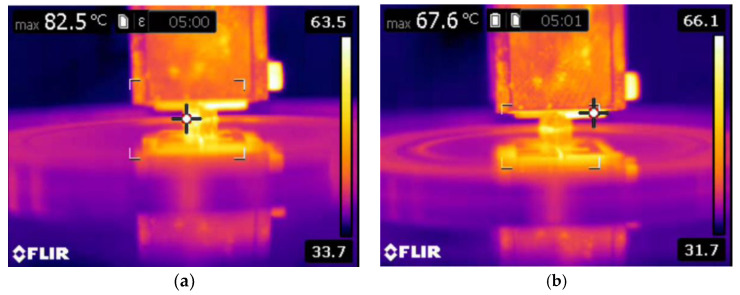
Temperature of the pin at 50 N loads at 5 min: (**a**) 5 wt.% and (**b**) 10 wt.%.

**Figure 14:**
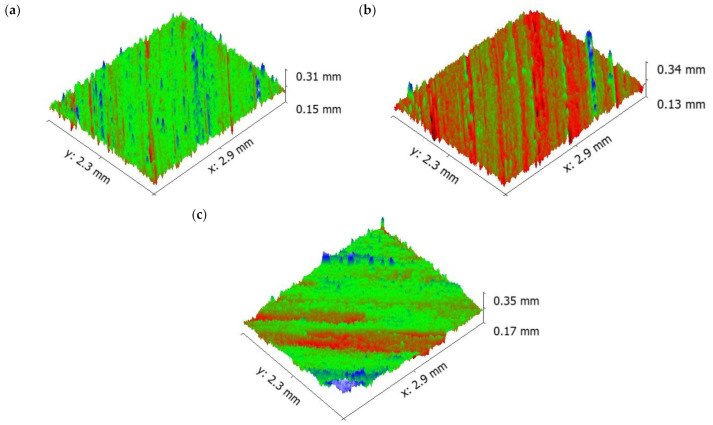
Worn surface topography of the 10 wt.% composite: (**a**) 0.75 m/s (**b**) 1.5 m/s (**c**) 2.25 m/s.

Figure 12 shows the results of the 10 wt.% reinforced composite. The contact area is still reduced because of the existence of more reinforced particulates on the surface of the pin material. Hence, the primary reinforcement particulates took more load than the matrix aluminium. In addition, these particulates were stronger and harder than the aluminium, therefore they were able to resist the applied load for a longer time, which could be the reason in the reduction of specific wear of the 10 wt.% reinforced composite material. At a higher reinforcement, the particulates not only resisted the wear but also started to wear the material of the counter surface. The primary reinforced particulates became fractured during the process; they were unable to penetrate into the counter surface but tried to penetrate into the aluminium matrix material. Hence, the particulates between the counter surface and pin material became powder particulates, which in turn led to the three-body wear mechanism. The latter helped to reduce the temperature between the counter surface and pin material, which could be one of the reasons for improving the wear resistance properties of the specimen. The temperature between the counter surface and pin material is shown in Figure 13. A similar observation was also made by Baradeswaran et al. [9]. The wear behaviour of the wear composites and hybrids varied with the load, and it was indicative of Archard’s law as well as significantly lower in the case of hybrid composites [31]. In the 10 wt.% specimen, the temperature between the counter surface and pin material was lower than the 5 wt.% reinforced and pure aluminium material (Figure 13). Similarly, the groove formed in the worn surface (Figure 14) was narrow and wide and the depth also low.

The SEM image of the 10 wt.% reinforced composite at sliding velocity 2.25 m/s and applied load at 50 N (Figure 12c) shows that the plastic flow in the material along with the small cavity indicates severe wear in the layer of the composite material. Severe plastic deformation in the worn surface led to the initiation of cracks. The delimitation occurred due to the damage in the layer of the composite. The force acting in the normal and tangential direction triggered the nucleation and propagation of cracks under the subsurface due to the contact of hard asperities. The propagation of cracks grew further and joined with nearby cracks and, in turn, it started to detach the small layer from the specimen surface. At higher load, most of the worn surfaces contained considerable delimitation along with long small plastic flow. A similar pattern was observed by Guleryuz et al. [44].

**Figure 15:**
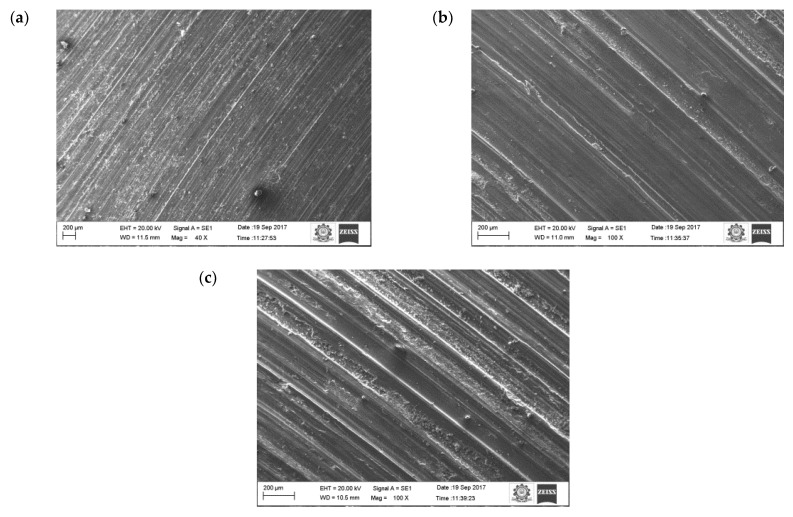
Worn surface of the hybrid composite: (**a**) 0.75 m/s (**b**) 1.5 m/s (**c**) 2.25 m/s.

In the case of hybrid composites, there was no plastic flow formation (Figure 15), only ridges and grooves running along the sliding direction were observed. This was attributed to the strengthening mechanism of the matrix material along with the primary and secondary reinforcements. Dislocation density could be the reason for strengthening the matrix and reinforcements. An increase in the primary and secondary reinforcements increased the dislocation density. The reason for the increase in dislocation density was due to the stress (thermal) developed between the aluminium and particulate interface. The thermal stress developed due the thermal characteristics of the matrix and the primary and secondary reinforcement materials. The thermal stress generated during the fabrication process delayed the sliding wear behaviour of the composite [45].

**Figure 16:**
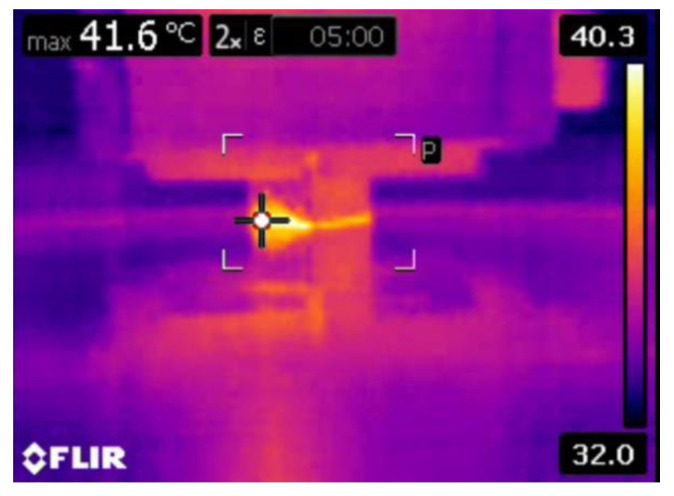
Temperature of the hybrid composite pin at 50 N loads at 5 min.

**Figure 17:**
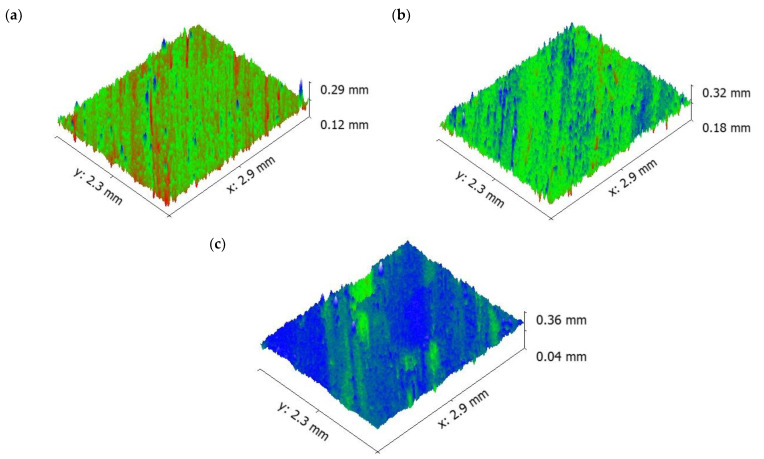
Worn surface topography of the hybrid composite: (**a**) 0.75 m/s (**b**) 1.5 m/s (**c**) 2.25 m/s.

Moreover, the aforementioned, shape and size of the primary and secondary reinforcement also played an important role in the dry sliding wear process. Most of the secondary reinforcements’ shape was regular when compared to the primary reinforcements. The regular shaped particulates were very strong in withstanding the tangential force generated during the dry sliding wear process. As such, the secondary reinforcement particulates in the pin retained its position for a longer time and reduced the contact between the counter surface and the pin material. As a result, there was no direct contact between the counter surface and pin material; this small gap was sufficient enough to remove the heat from the zone. It is evident from the thermal image (Figure 16) that the temperature of the counter surface and pin was lower than the unreinforced and primary reinforced composites for the same sliding condition and normal load. The surface roughness of the hybrid composite was also very low when compared to the unreinforced and primary reinforced composites (Figure 17). The intact behaviour of the reinforcement with matrix materials allowed only the abrasion wear mechanism, and no plastic deformation and delimitation, which in turn reduced the peaks and valleys in the worn surface.

The reason for the reduced wear rate was due to the shape of the secondary reinforcements. The primary reinforcements were not uniform in shape and subjected to severe fracture, even for small loads, which led the particulates to pull out from the matrix alloy. The SEM image of the worn surface (Figure 12c) illustrates this pulling out of some of the particulates from the matrix material.

### 3.4. Friction Coefficient

**Figure 18:**
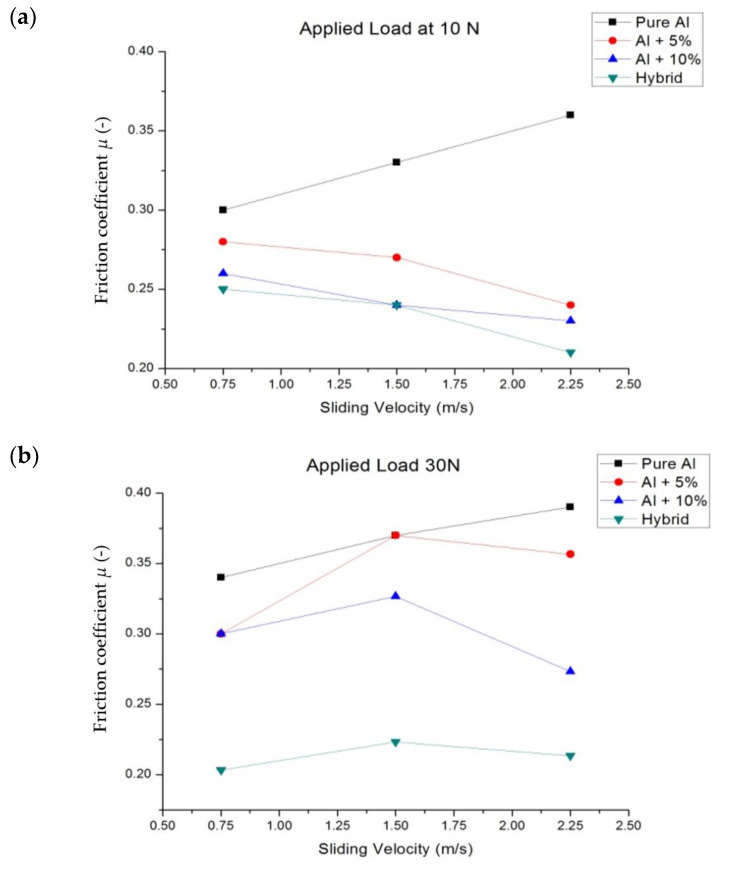

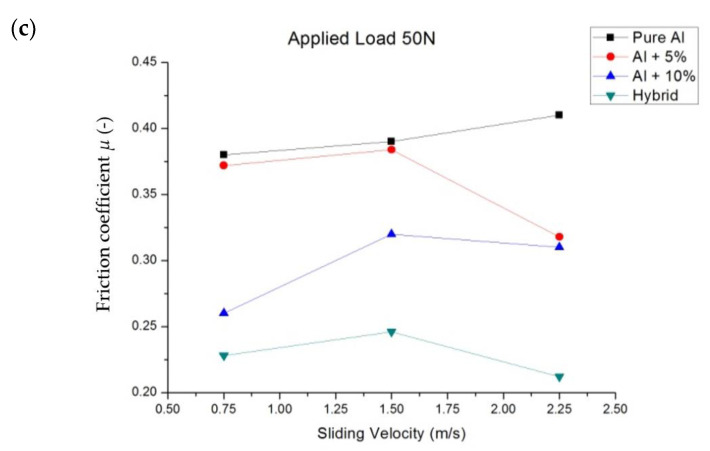
Friction coefficient: (**a**) 10 N (**b**) 30 N and (**c**) 50 N.

The effect of the sliding speed on the friction coefficient is shown in Figure 18. The trend of the friction coefficient is similar to that of specific wear rate. It is observed that the increase in sliding speed increases the friction coefficient for the pure aluminium material and is reduced for the primary reinforced and hybrid composites. In general, an increase in sliding velocity decreases the friction coefficient—high velocity results in high heat of the surface. Because of this formation of a small oxide layer at the roughness contacts, that layer averted contact with the surface, which in turn reduced the friction coefficient. However, in this case, there are several wear lines (Figure 7c), indicating that the metal softening occurred during the dry sliding process. This could be the reason for the increase in friction coefficient at higher sliding velocity.

It is worth noting that the friction coefficient of pure aluminium was higher than that for the primary reinforced and hybrid composites, which was attributed to the formation of an alumina oxide layer between the counter surface and pin material. During the wear process, the reinforced particulates that came out from the matrix material contained a rich amount of alumina oxide (Table 1). Moreover, the aluminium also reacted with oxygen at an elevated temperature to form alumina oxide. This formed a strong layer which was good enough to reduce the shearing action, but at higher load these oxide layers were torn out and the friction coefficient started to increase drastically.

In the case of the hybrid specimen, the friction coefficient was slightly lower than the pure aluminium, 5 and 10 wt.% reinforced composites, and there might be two reasons for this. The first reason could be the formation of an alumina oxide layer along with the boron oxide layer. The secondary reinforcement removed from the pin material readily reacted with the environment to form boron oxide, and the oxides layer formed between the counter surface and pin material influenced its friction coefficient. The second reason could be the effect of porosity of the composites. At the 5 and 10 wt.%, the primary reinforced composites had more porosity than the hybrid composites. An increase in porosity also increased the friction coefficient. The average friction coefficient obtained by varying the load was 0.211, 0.2122, 0.214, respectively. That result was in agreement with the results obtained by Mazaheri et al. [46] but the friction coefficient was 0.340 for the hybrid composite and was approximately two times higher.

At a sliding velocity of 1.5 mm/s and higher load of 30 N and 50 N, the stable oxide layer formed during the wear process was broken and the reinforcement particulate pull-out was reported in SEM, leading to the increase in the friction coefficient equal to that of 0.75 mm/s. However, at a low load of 10 N, the friction coefficient decreased as the sliding velocity increased.

However, in some cases the temperature difference was more or less constant for the primary reinforced composites due to the existence of the mechanically mixed layer and small amount of porosity. The existence of porosity failed to transfer the temperature to the counter surface or pin material completely. In the case of the hybrid composites, the rise in temperature was less when compared to the primary reinforced specimen. Although the porosity in the specimen (hybrid) was less than the unreinforced and primary reinforced composites, there should be an increase in the friction coefficient. That the strong oxide layer, due to the alumina oxide and boron carbide, took a longer time to wear the oxide layer could be the reason for the reduction in the friction coefficient.

## 4. Conclusions

The following observations were made:

- The vacuum assisted stir casting process helped to fabricate the primary reinforced and hybrid composite with minimum porosity, i.e., less than 5% for the primary and 10% for the hybrid composite;
- The fabricated specimen had better properties than the pure aluminium. The effect of the 5 wt.% primary reinforcements raised the tensile strength of the specimen up to 20%, whereas a further increase in primary reinforcements reduced the tensile strength of the composites to 34%. The hybrid composites obtained better results than the 10 wt.% reinforced composites; the reduction of tensile strength is up to 30% only;
- The impact strength of the composites was similar to the tensile strength; the 5 wt.% reinforced composites increased the impact strength up to 15%, whereas the impact strength of the 10 wt.% primary reinforced and hybrid composites decreased to 57 and 49%, respectively;
- The hardness of the fabricated specimen increased with the addition of primary and secondary reinforcement when compared to the pure aluminium;
- The specific wear rate of the hybrid composites was lower than the pure aluminium and primary reinforced composites for all cases of sliding velocity and normal load;
- The friction coefficient on pure aluminium increased with the increase in sliding velocity, whereas the friction coefficient of the primary and hybrid composites decreased with an increase in sliding velocity;
- The worn surface of the pure aluminium had a plastic deformation zone and delaminated surface at higher load, whereas the primary and hybrid composites contained only narrow grooves due to the abrasion wear mechanism;
- The surface topography of the pure aluminium and reinforced composites had a rougher surface, whereas the hybrid composites had a smoother surface with regularly spaced peaks and valleys.

## Figures and Tables

**Table 1:** Sillimanite chemical composition.

Elements	Al_2_O_3_	SiO_2_	TiO_2_	ZrO_2_	Fe_2_O_3_	CaO	MgO
**wt.%**	55–58	37–38	0.25–0.64	2.20–3.50	0.30–0.40	0.01–0.02	0.03–0.04

**Table 2:** Properties of aluminium, sillimanite and B_4_C.

Properties	Aluminium	Sillimanite	B_4_C
Density (g/cm^3^)	2.70	3.24	2.30–2.52
Hardness (kg/mm^2^)	17.02	7	3000
Elastic modulus (GPa)	80	159	362–427
Melting point (°C)	660	1850	2450
Coefficient of thermal expansion (10^−6^/°C)	2.20	4.50	3.2

## Data Availability

Data is contained within the article.
